# Mental health presentations to acute psychiatric services: 3-year study of prevalence and readmission risk for personality disorders compared with psychotic, affective, substance or other disorders

**DOI:** 10.1192/bjo.2018.72

**Published:** 2018-12-21

**Authors:** Kate L. Lewis, Mahnaz Fanaian, Beth Kotze, Brin F. S. Grenyer

**Affiliations:** Associate Research Fellow, School of Psychology, University of Wollongong, Australia; Lecturer, School of Nursing, University of Wollongong, Australia; Director, Mental Health, Cumberland Hospital, Australia; Professor of Psychology, School of Psychology, University of Wollongong, Australia

**Keywords:** Inpatient treatment, hospital admissions, personality disorders, borderline personality disorder

## Abstract

**Background:**

The relative burden and risk of readmission for people with personality disorders in hospital settings is unknown.

**Aims:**

To compare hospital use of people with personality disorder with that of people with other mental health diagnoses, such as psychoses and affective disorders.

**Method:**

Naturalistic study of hospital presentations for mental health in a large community catchment. Mixed-effects Cox regression and survival curves were generated to examine risk of readmission for each group.

**Results:**

Of 2894 people presenting to hospital, patients with personality disorder represented 20.5% of emergency and 26.6% of in-patients. Patients with personality disorder or psychoses were 2.3 times (95% CI 1.79–2.99) more likely than others to re-present within 28 days. Personality disorder diagnosis increases rate of readmission by a factor of 8.7 (s.e. = 0.31), marginally lower than psychotic disorders (10.02, s.e. = 0.31).

**Conclusions:**

Personality disorders place significant demands on in-patient and emergency departments, similar to that of psychoses in terms of presentation and risk of readmission.

**Declaration of interest:**

None.

## Prevalence of personality disorder

Epidemiological studies estimate that the prevalence of personality disorder is about 6–6.5% of the general population.^1^^,^^2^ Personality disorders can cause significant interpersonal and intrapersonal difficulties, disruptions in social and occupational functioning^3^^–^^5^ and can have high societal costs.^6^ Many people with personality disorder have involvement with mental health services, with some having extensive histories of out-patient and in-patient care,^7^^–^^9^ as well as high rates of pharmacotherapy use,^10^ although there are known cultural and gender differences in accessing services.^11^

In general, people with personality disorder are at significantly higher risk of mortality than the general population.^12^ They are also at higher risk of suicidal behaviours and self-harm,^13^ and rates of comorbidity of other mental health conditions such as mood, anxiety and substance use disorders are also high.^14^ Some people with personality disorders evidence crisis-prone, risky and impulsive behaviours – which can result in frequent presentations to emergency departments and admission to in-patient hospital units.^9^^,^^15^ Disorders such as schizophrenia, depression and bipolar disorder are also highly prevalent in mental healthcare settings. Few studies, however, have directly compared the hospital-based mental health presentations and readmissions of different disorder groups.

## Personality disorder and service use

Compared with people with depression, people with personality disorder are more likely to have engaged in multiple treatment modalities,^16^ and are more likely to have extensive histories of in-patient and out-patient treatment.^7^ Specifically, recent studies have shown that having a personality disorder is associated with a greater number of hospital admissions,^17^ and there is evidence to suggest that people with borderline personality disorder are more likely than any other disorder group to re-present to emergency or be readmitted to an in-patient mental health unit.^18^ One study of emergency department presentations demonstrated that people with personality disorder were more likely to make more repeated visits and spend longer in the emergency department than people with other mental health diagnoses. Similarly, they were more likely to be brought in by police, after hours and to be discharged back home.^19^ Interestingly, this study reported only 6% of people presenting to the emergency department had a diagnosis of personality disorder, which is significantly lower than other studies.^8^^,^^20^

Hospital admissions for people with personality disorder are not generally recommended within current treatment guidelines unless the patient is at high risk for suicide or medically serious self-harm. In any case, it is recommended that the stay should be brief and focused on the current risk.^21^^,^^22^ Despite this, Shoka and colleagues^23^ examined the length of stay in a mental health in-patient unit for 137 discharges and reported that the median length of stay for borderline personality disorder was 10 days, which was longer than drug addictions (6 days) and adjustment disorders (5 days), but shorter than psychoses (28 days) and mood disorders (14 days). Other studies have examined the impact of comorbid personality disorder on service use, and reported that having a comorbid diagnosis of personality disorder alongside a primary diagnosis of another serious mental illness is significantly associated with higher use of in-patient services and involuntary admissions.^24^ An important variable measured by hospital administrators is risk of short-term readmission^25^^,^^26^ as this may be considered a result of inadequate care or discharge into the community.

## The current study

Although it is well known that people with personality disorders are high users of health services, there are few comparative studies and no studies of relative risk of readmission, with much of the existing literature focusing on borderline personality disorder. In this study, we examined the mental health hospital use (in-patient and emergency) and rates of readmission of people with any personality disorder compared with other major mental health diagnostic groups across a representative community catchment.

## Method

### Setting

The study setting was a population catchment of 270 050 people with two major hospitals.^27^ The site chosen was unremarkable in that it was representative of a typical hospital network serving its local community. The community catchment was representative of the broader Australian community at the time, with a similar median age (37.9 *v.* 37.2 nationally), the same gender distribution (50.23% females), and the same proportion of Aboriginal and Torres Strait Islanders (2.6%) (ABS, 2011). There were no private or alternative hospital beds locally for mental health patients at the time, and because of its geographic location, outflows to other health services were reported to be minimal. Between the two local hospitals were two emergency departments and eight mental health in-patient units. In-patient facilities included adolescent, rehabilitation, geriatric and psychiatric emergency care, and four general mental health in-patient units. Community teams were available for crisis, acute and ongoing case management services.

### Data sources

All mental health in-patient and emergency admissions (measured as separations) were studied across 36 months. The term ‘separation’ is used here to refer to separation from the hospital system through discharge by medical staff, by self against medical advice, discharge to other private unit or home care, or death. Data included admission and discharge dates, demographic information and diagnostic information. All patients were routinely diagnosed by mental health professionals trained in a structured, health service protocol^28^ to derive ICD-10 primary diagnosis.^29^ Administratively, diagnoses are based on the Australian Refined Diagnosis Related Groups model (AR-DRG) of consumer classification.^30^ For some patients their diagnosis differed between admissions, therefore clinical experts assigned the final primary diagnosis. For example, people with personality disorders may have had their diagnosis delayed, having presented with complex diagnostic histories prior to being given a formal diagnosis of personality disorder.^18^^,^^31^

Data was extracted by the records manager at the hospital from the hospitals electronic medical record keeping system. The data extracted was data routinely collected by the service, and was provided as de-identified information. Information was collected in accordance with health service guidelines, and this study was reviewed and approved by the institutional review board (HE10371).

### Statistical analysis

Data obtained was in a format allowing separation-level analysis and rotation to patient level by anonymous unique identifiers. Same individual separations were identified, and grouped in chronological order allowing analysis of indices such as total number of separations, and total number of bed days over the study period for each patient. Continuous admissions for a single mental health episode, including interhospital or unit transfers were identified using the stay identifier number and analysed as a single event.

The AR-DRG classifications for each separation were pooled into five larger diagnostic groups (personality disorders, psychotic disorders, affective disorders, substance disorders, self-harm) to allow meaningful comparisons of groups of disorders that have similarities in their symptomatic presentation and typical treatment trajectories (supplementary Table 1 available at https://doi.org/10.1192/bjo.2018.72). The AR-DRG classifications (based on the ICD-10) align with those in the DSM-IV-TR, published by the American Psychiatric Association.^32^

Patients that did not fit best into the main diagnostic groups were classified as ‘other’. The ‘other’ classification comprised diagnoses that were found to be of low prevalence in this setting, including eating and obsessive–compulsive disorders, childhood mental disorders and dementia. In some instances, clinicians reported the diagnosis as medical (i.e. cerebrovascular disorders, seizures), but because of concurrent mental health problems, treatment was administered by mental health teams. These instances were also classified as ‘other’. Self-harm injuries and poisonings, not accompanied by any other diagnosis were grouped as self-harm only.

The cases of three participants were removed. One because of data entry error, whereby the length of stay was unable to be accurately determined, and the other two because of atypical presentations; investigation of these patients indicated that they were being kept in hospital beds for long stays (almost across the entire duration of our study) as a result of an inability to place them into suitable longer stay rehabilitation, forensic or supported community care settings. They were therefore not representative examples of how in-patient mental health stays occur or are used, and distort the data risking creating a misleading picture of the typical use and function of mental health in-patient beds.

At the patient level, this paper reports the average number of hospital separations, the stay details (i.e. bed days) and demographic characteristics. Between-group comparisons were done using ANOVA for continuous data, and χ^2^ for categorical and binary data. For readmissions, we considered the number of days from each discharge to readmission as a time-to-event variable with censoring and analysed with a mixed-effects Cox regression model. This model is an extension of the Cox proportional hazard model, which incorporates random effects to account for within-cluster homogeneity,^33^ in this case individuals. This model took into account all admissions and readmissions for each individual. This analysis was performed in the R statistical programming language using the R Studio program, the ‘coxme’ package, and the ‘survminer’ package to develop survival plots.

## Results

### Participants

In the study period there were 6486 episodes of care for 2894 patients. Following rotation of data to patient level, the total in-patient sample was 2833 individuals, and the emergency department sample was 1104 individuals, however, some of the participants had presentations to both in-patient and emergency departments within the study period. Specifically, 1043 had at least one in-patient and emergency episode, 1790 had at least one in-patient episode only and 61 had at least one emergency department episode only. When emergency presentations and in-patient stays are combined for each individual, the average number of hospital separations per patient for this sample was 2.25 (s.d. = 2.22), with a range of 1–36. The 2833 in-patients had an average of 1.73 (s.d. = 1.56, median 1.0) in-patient stays in the study period of 36 months, ranging 1–23. Of the 1104 who presented to an emergency department, the average number of separations was 1.47 (s.d. = 1.22, median 1.0), ranging 1–20.

**Fig. 1:**
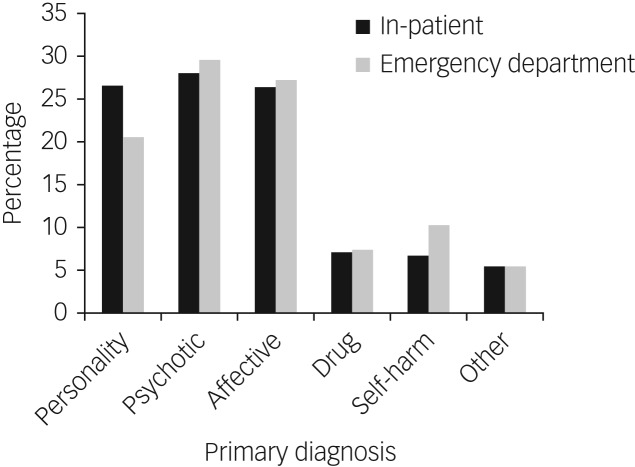
Primary diagnosis for the in-patient sample (*n* = 2833) and the emergency department sample (*n* = 1104).

Psychotic disorder was the most prevalent primary diagnosis in the emergency department sample (*n* = 326, 29.5%), followed by affective (*n* = 300, 27.2%) and personality disorder (*n* = 226, 20.5%). A total of 81 patients (7.3%) had a primary substance diagnosis and 113 (10.2%) self-harm only. In addition, 58 (5.3%) had no diagnoses within these groups (Fig. 1). Because analysis of the ‘other’ group would not yield clinically meaningful results, they were excluded from further analyses.

The in-patient sample followed a similar pattern. Psychotic disorder was the most prevalent primary diagnosis in the in-patient sample (*n* = 792, 28.0%), followed by personality disorder (*n* = 754, 26.6%) and affective disorder (*n* = 748, 26.4%). There were 197 patients with a primary substance disorder (7.0%) and 189 with self-harm only (6.7%). A total of 153 had diagnoses that did not fall into any of the main diagnostic groups (5.4%).

Table 1 gives the demographic characteristics. Age differed across diagnostic groups for both the in-patient (*F*(4) = 42.17; *P* < 0.001) and emergency department samples (*F*(4) = 20.32; *P* < 0.001). Average ages for diagnostic groups within the in-patient and emergency department samples followed a similar trend – patients with substance or personality disorder were younger, and affective disorders were older. Gender distribution was relatively even (in-patient 48.2% female, emergency department 48.4% female), yet varying considerably across disorder groups for both in-patient (*F*(4) = 45.57; *P* < 0.001) and emergency department samples (*F*(4) = 18.88; *P* < 0.005).

**Table 1:** Demographic characteristics and significance tests for patients across primary diagnostic groups for the in-patient sample (*n* = 2833) and for the emergency department sample (*n* = 1104)^a^

	Overall		Personality		Psychotic		Affective		Substance		Self-harm		*F*	χ^2^	*P*
*In-patients, n^b^*	2833		754		792		748		197		189				
Age, years: mean (s.d.)	40.76	(16.67)	36.61	(14.17)	41.59	(14.88)	46.00	(18.84)	34.14	(12.96)	39.55	(17.45)	42.17	–	<0.001
Women, *n* (%)	1365	(48.2)	363	(48.1)	330	(41.7)	411	(54.9)	71	(36.0)	109	(57.7)	45.57	–	<0.001
Marital status, *n* (%)														219.00	<0.001
Married	692	(24.9)	185	(24.9)	106	(13.9)	267	(36.2)	27	(13.9)	65	(34.9)	–	–	–
Single	1485	(53.5)	400	(53.8)	526	(69.0)	272	(36.9)	131	(67.5)	77	(41.4)	–	–	–
Divorce/separated	476	(17.2)	142	(19.1)	104	(13.6)	143	(19.4)	34	(17.5)	35	(18.8)	–	–	–
Widowed	121	(4.4)	16	(2.2)	26	(3.4)	56	(7.6)	2	(1.0)	9	(4.8)	–	–	–
Australian born, *n* (%)	2363	(84.0)	664	(88.5)	641	(81.7)	600	(80.5)	177	(90.8)	156	(83.4)	–	28.11	<0.001
English language, *n* (%)	2767	(98.0)	743	(98.8)	764	(97.1)	734	(98.4)	196	(99.5)	187	(98.9)	–	9.96	0.041
*Emergency department, n^c^*	1104		226		326		300		81		113				
Age, years: mean (s.d.)	42.08	(16.43)	38.66	(13.72)	41.79	(14.50)	47.76	(18.22)	34.31	(12.66)	37.33	(16.16)	20.32	–	<0.001
Women, *n* (%)	534	(48.4)	112	(49.6)	139	(42.6)	153	(51.0)	30	(37.0)	71	(62.8)	18.88	–	<0.001
Marital status, *n* (%)															
Married	273	(25.1)	62	(27.8)	44	(13.8)	105	(35.5)	12	(15.2)	36	(32.1)	–	–	–
Single	575	(52.9)	116	(52.0)	223	(70.1)	101	(34.1)	53	(67.1)	55	(49.1)	–	–	–
Divorce/separated	187	(17.2)	40	(17.9)	43	(13.5)	60	(20.3)	14	(17.7)	19	(17.0)	–	–	–
Widowed	51	(4.7)	5	(2.2)	8	(2.5)	30	(10.1)	0	–	2	(1.8)	–	–	–
Australian born, *n* (%)	919	(83.7)	198	(88.4)	270	(83.3)	240	(80.0)	73	(91.3)	91	(81.3)	–	10.55	0.03
English language, *n* (%)	1074	(97.5)	221	(98.2)	312	(96.0)	293	(97.7)	81	(100.0)	112	(99.1)	–	7.04	0.13

a. Missing data. In-patient sample: marital status *n* = 59, Australian born *n* = 19, language *n* = 10. Emergency department sample: marital status *n* = 18, Australian born *n* = 6, language *n* = 2.

b. For the in-patient sample there were 58 (5.3%) individuals who had no diagnoses within these groups.

c. For emergency department sample there were a total of 153 individuals who had diagnoses that did not fall into any of the main diagnostic groups (5.4%).

### Readmissions

**Fig. 2:**
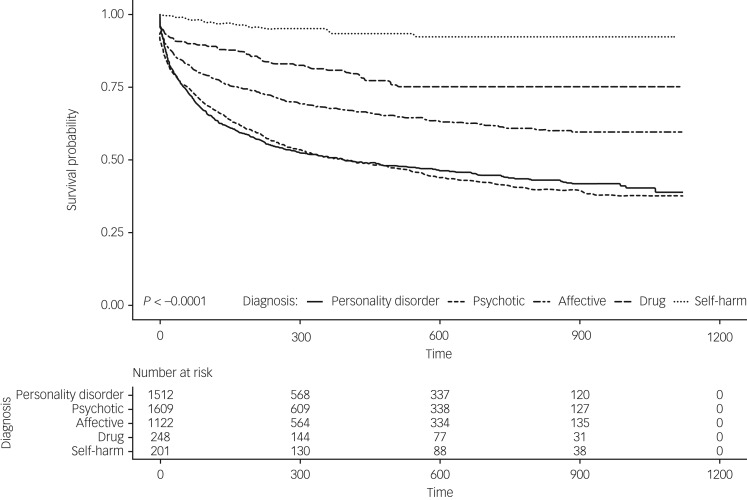
Survival curve for readmission to an in-patient unit within the study period.

The mixed-effects Cox regression model (χ^2^ = 681.39 (d.f. = 8), *P* < 0.001) showed the time to readmission differed according to diagnostic group (log rank χ^2^ = 681.39 (d.f. = 8), *P* < 0.001) after accounting for age and gender. Mean time to readmission for personality disorder was 309.09 days (s.e. = 8.29, 95% CI 292.84–325.35, median 177). Mean time to readmission (in days) was shortest for psychotic disorders (mean 306.70, s.e. = 7.91, 95% CI 291.18–322.22, median 186) and longest for self-harm (mean 512.06, s.e. = 23.81, 95% CI 465.11–559.02, median 488.00). Consistent with this, analyses showed that psychotic disorder increased the hazard of readmission more than any other disorder group (hazard ratio (HR) = 10.02, s.e. = 0.31, *P* < 0.001), followed by personality disorder (HR = 8.73, s.e. = 0.31, *P* < 0.001), affective disorder (HR = 5.71, s.e. = 0.31, *P* < 0.001), substance (HR 3.35, s.e. = 0.34, *P* < 0.001), and self-harm (HR = 0.96, s.e. = 0.42, *P* = 0.091). Figure 2 shows the survival curve for readmission to an in-patient unit within the study period. Note the number-at-risk table represents all admissions for each diagnostic group, not individuals.

The number-at-risk table represents all admissions for each diagnostic group, not individuals.

Of all patients who had an in-patient stay in the study period, 12.0% (*n* = 340) had been readmitted to at least one of the two hospitals within 28 days of discharge. More than two-thirds of these people had either primary personality disorder (36.5%) or a primary psychotic disorder (37.6%). More than one-fifth (21.5%) had a primary affective disorder, 4.1% substance disorder and 0.3% self-harm. The average number of 28-day readmissions for all patients with personality disorder was 0.85 (s.d. = 1.62). This rate was significantly larger (*F*(4) 5.189, *P* < 0.001) than the average number of 28-day readmissions for psychotic disorder (mean 0.53, s.d. = 0.94), affective disorder (mean 0.45, s.d. = 0.92), substance disorder (mean 0.44, s.d. = 0.56) or self-harm (mean 0.09, s.d. = 0.30). When pooled together, patients with personality disorder or psychotic diagnosis were 2.3 (95% CI 1.79–2.99, χ^2^ = 43.07; *P* < 0.001) times more likely to have at least one readmission within 28 days of discharge than those who had either an affective, substance or self-harm disorder.

### Admissions and bed days

Total number of in-patient admissions differed between diagnostic groups (*F*(4) = 31.15; *P* < 0.001), as did the number of emergency department presentations (*F*(4) = 12.35; *P* < 0.001) (Table 2). Specifically, of the five main diagnostic groups, patients with personality disorder had a larger mean number of admissions than all other diagnostic groups, except the psychotic group. The number of emergency department presentations was also highest for personality disorder and the psychotic group. Total number of bed days also differed between diagnostic groups (*F*(4) = 71.45; *P* < 0.001). Patients in the psychotic group had a considerably greater average number of bed days over the study period (mean 55.19, s.d. = 81.16), and those in the self-harm group had the fewest (mean 8.19, s.d. = 11.46). The mean number of bed days for patients with a personality disorder in this study period was 18.28 (s.d. = 37.46) (Table 2).

**Table 2:** Total number of emergency department presentations for 1104 emergency department patients, and in-patient stays and total number of bed days for 2833 in-patients, over the 3-year study period, stratified by diagnostic group

	In-patient admissions			Bed days in in-patient unit			Emergency department presentations		
	Mean (95% CI)	s.d.	Median	Mean (95% CI)	s.d.	Median	Mean (95% CI)	s.d.	Median
Personality	2.03 (1.87–2.18)	2.20	1.0	18.28 (15.60–20.96)	37.46	6.0	1.79 (1.56–2.02)	1.77	1.0
Psychotic	2.05 (1.94–2.16)	1.56	1.0	55.19 (49.53–60.85)	81.16	29.0	1.69 (1.53–1.85)	1.44	1.0
Affective	1.52 (1.44–1.59)	1.06	1.0	25.88 (23.27–28.49)	36.36	13.0	1.31 (1.23–1.39)	0.71	1.0
Substance	1.27 (1.15–1.38)	0.80	1.0	10.81 (8.94–12.67)	13.27	7.0	1.12 (1.04–1.21)	0.40	1.0
Self-harm only	1.06 (1.03–1.10)	0.27	1.0	8.19 (6.54–9.83)	11.46	4.0	1.07 (1.02–1.12)	0.29	1.0

## Discussion

### Main findings

This study examines patterns of hospital use for people with personality disorder and those with other mental health problems. We analysed all hospital admissions within a 3-year study period within a large community catchment. In this sample, people with personality disorders and psychotic disorders presented to hospitals most frequently, and were more likely to be readmitted sooner. These two groups along with individuals with affective disorders represented about two-thirds of all mental health admissions.

Individuals with personality disorder in this study had on average 18.3 bed days in the 3-year study period, equating to approximately 6 days per year. Although they spent fewer days in hospital than those with psychotic disorders and affective disorders, they were admitted to hospital more frequently than patients in affective, substance and self-harm groups. Having a personality disorder was also associated with more short-term readmissions, consistent with Shoka and colleagues.^23^

Survival analysis demonstrated that people with psychotic disorders were more likely to be readmitted earlier, followed by the personality disorder, affective, substance and self-harm groups. Compared with personality disorder, at any given time, there was a significantly higher risk of readmission for psychotic disorders, but a significantly lower risk of readmission for affective and substance disorders, after controlling for age and gender. The risk hazard for self-harm was not significant. This is likely because of the small sample size, and the small number of readmissions for this group.

### Directions for future studies

People with either a psychotic or personality disorder were 2.3 times more likely than other diagnostic groups to be readmitted to hospital within 28 days of discharge. Further studies exploring the nature and reasons for rapid readmissions would make a significant contribution to the literature. Specifically, determining whether there was a lack of follow-up care because of non-adherence by patients or systemic delays, would be valuable information for people working within in-patient unit, and relevant policymakers.

In this study, we found diagnosis of personality disorder was equally represented between male and female patients. Although this is consistent with epidemiological studies,^2^ studies report that women with personality disorder are more likely to seek help than men, particularly for repeated self-harming behaviours.^5^ The complexities of gender and personality disorder in treatment settings requires further understanding and exploration, particularly with the consideration of specific types of personality disorder (for example borderline, narcissistic), which unfortunately is beyond the scope of this paper.

This is a study of one health service in New South Wales, Australia. Although the health service at the time was unremarkable from others in the state in terms of hospital and out-patient services, the generalisability of these findings to other services in Australia and internationally, should be considered in light of local policies around admissions to hospital, availability of hospital beds and quality of out-patient services. Also worth noting is that although in general hospital admissions for people with personality disorders are not recommended, both the National Health and Medical Research Council and National Health and Care Excellence guidelines suggest that in some cases it is necessary,^21^^,^^34^ and some individuals with borderline personality disorder show positive long-term results from in-patient treatment, particularly for people with complex comorbidity.^35^ Future studies would benefit from further investigation about treatment received within their in-patient stay, and further determination of whether the hospital stay was helpful or not or whether it could have been prevented particularly in the case of short-term readmissions.

It should also be noted, that in Australia many people with personality disorder do not access public mental health services, with some seeking treatment privately, others through non-government organisations and the majority of people not accessing treatment at all.^36^ Thus, the findings of this paper only reflect public hospital use of people who access this treatment, and those whose symptoms tend to be the most severe.

### Limitations

This study has limitations. First, we were not able to ascertain whether individuals had been admitted or presented in an emergency to a hospital outside this region, nor were we able to identify any that moved out of the region within the study period. Consequently, the number of mental health separations may be underestimated. Second, self-harm is often an acute feature of personality disorder.^37^ It is possible that some people in the self-harm group may have personality disorder, with a diagnosis not yet assigned. In this study, self-harm episodes, not accompanied by any other disorder were considered as stand-alone, despite it being one criterion of borderline personality disorder.^38^ Thus, it is possible the data underestimates the prevalence of personality disorder.

Finally, the single classification of personality disorder reported here may represent a heterogeneous group. It is expected variations in personality disorder traits would influence whether individuals present for treatment and the types of services to which they present. Unfortunately, specific types of personality disorder (for example borderline) were not coded and thus not available for study. Future studies examining in-patient hospital use for specific groups of personality disorder are required.

### Diagnosing personality disorder

There are also several clinical issues related to diagnosing personality disorder that extend beyond the scope of this study. In clinical practice, the diagnosis of personality disorder tends to be conservative and delayed, and it is also suggested personality disorder is often underrecognised by clinicians.^18^^,^^39^ This may be because of the ready recognition of depression and anxiety in an individual's presentation,^18^^,^^40^ and a degree of hesitation to diagnose personality disorder because of stigma. Presentation upon admission to in-patient units and emergency departments generally involves escalated crisis-prone symptoms and suicidal risk, meaning diagnosis may not be clearly obtained. Although in this study we used discharge diagnoses, which tend to be more accurate as they benefit from longer observation of the patient, it is possible personality disorder diagnoses were underestimated. Diagnostic comorbidity is also high in people with personality disorder.^41^ Future studies should examine the effect of comorbidities on in-patient treatment use and outcome.

### Implications

Despite the limitations, this study clearly indicated that some people with personality disorder present frequently to emergency department and in-patient units for care, and represent a significantly large proportion of mental health patients in these settings. In-patient hospital stays are costly, and although sometimes necessary for treatment of injury or suicidality, evidence-based guidelines for personality disorders indicate psychotherapy in the community as the treatment of choice.^21^^,^^34^

This study demonstrates that personality disorders place significant demands on the health system, with a similar impact to that of psychoses in terms of frequency of presentations and risk of short-term readmissions. In light of these findings, and considering people with personality disorders represent more than one-fifth of all mental health in-patients, this study highlights the value of further review of the management of people with personality disorders by hospital systems and governing bodies.^42^ Specifically, exploring strategies to intervene in the community setting and before the escalation in crisis to admission to hospital, as well as evaluating the benefits of rapid follow-up and stepped-care treatment planning programmes in the community after discharge from hospital, would make a valuable contribution to the field.^43^ Future research may further examine existing pathways in and out of in-patient units to further understand the reasons why people with personality disorders are presenting, and how they are being managed. The benefits of such initiatives may result in improvement in patient outcome and reduction of burden to the hospital system.

## Supplementary material

For supplementary material accompanying this paper visit http://dx.doi.org/10.1192/bjo.2018.72. click here to view supplementary material
