# Supplementary material for: Mental health presentations to acute psychiatric services: 3-year study of prevalence and readmission risk for personality disorders compared with psychotic, affective, substance or other disorders
Source: BJPsych Open. 2018 Dec 21;5(1):e1. doi: 10.1192/bjo.2018.72 (PMC6343119; doi:10.1192/bjo.2018.72)
Supplement: Supplementary file 1 [file S2056472418000728sup001.docx]

**Supplemental Table 1.** Australian Refined Diagnosis-Related Groups (AR-DRG) based on ICD-10 structured clinical interviews on admission to inpatient or emergency department.

| Diagnostic group | AR-DRG diagnoses |
| --- | --- |
| Affective  (including major depression) | ‘Major affective disorders’; ‘Other affective and somatoform disorders’ |
| Personality disorder | ‘Personality disorder and acute reactions’ |
| Psychotic (including schizophrenia) | 'Schizophrenia disorders’; ‘Paranoia and Acute Psych Disorder (with or without catastrophic or severe consequences, or mental health legal status);’ ‘Schizophrenia (disorder)’; ‘Acute schizophrenic episode’; ‘Chronic schizophrenia’; ‘Disorganised schizophrenia’; ‘Psychotic disorder’. |
| Substance  (including alcohol dependence) | ‘Drug intoxication and withdrawal’; ‘Alcohol intoxication and withdrawal’; ‘Alcohol use disorder and dependence’; ‘Other drug use disorder and dependence’; ‘Opioid drug use disorder and dependence’; ‘Alcohol withdrawal syndrome’; ‘Admitted to alcohol detoxification centre’; ‘Feeling intoxicated’; ‘Alcoholism’; 'Drug overdose (disorder)’; ‘Alcohol dependence’. |
| Self-harm (not accompanied by another primary disorder category) | 'suicidal intent' ‘injuries, or poisoning, or toxic effects of drugs or other substances or psychotropic agents (with or without catastrophic or severe consequences or ventilator support)' |
| Other | ‘Dementia and other chronic disturbances of cerebral function’; ‘Anxiety disorders’; ‘Delirium (with or without catastrophic consequences)’; Degenerative nervous system disorders (with or without catastrophic consequences)’; ‘Eating and obsessive-compulsive disorders’; ‘Childhood mental disorders’; Other instances where the diagnosis reported was medical (ie. postpartum and antenatal problems, cerebrovascular disorders, seizures), but due to concurrent mental health problems, was classified by clinicians as a mental health admission/separation. |
